# LC-MS/MS-Based Profiling of Tryptophan-Related Metabolites in Healthy Plant Foods

**DOI:** 10.3390/molecules25020311

**Published:** 2020-01-13

**Authors:** Sara Vitalini, Michele Dei Cas, Federico Maria Rubino, Ileana Vigentini, Roberto Foschino, Marcello Iriti, Rita Paroni

**Affiliations:** 1 Department of Agricultural and Environmental Sciences, Università degli Studi di Milano, 20133 Milan, Italy; sara.vitalini@unimi.it; 2 Department of Health Sciences, Università degli Studi di Milano, 20142 Milan, Italy; michele.deicas@unimi.it (M.D.C.); federico.rubino@unimi.it (F.M.R.); rita.paroni@unimi.it (R.P.); 3 Department of Food, Environmental and Nutritional Sciences, Università degli Studi di Milano, 20133 Milan, Italy; Ileana.vigentini@unimi.it (I.V.); roberto.foschino@unimi.it (R.F.)

**Keywords:** Tryptophan, Tryptamine, indole compounds, matrix extraction, soya, Pumpkin, Sesame, Spirulina, nutraceuticals, functional foods, mass spectrometry

## Abstract

Food plants contain hundreds of bioactive phytochemicals arising from different secondary metabolic pathways. Among these, the metabolic route of the amino acid Tryptophan yields a large number of plant natural products with chemically and pharmacologically diverse properties. We propose the identifier “indolome” to collect all metabolites in the Tryptophan pathway. In addition, Tryptophan-rich plant sources can be used as substrates for the fermentation by yeast strains to produce pharmacologically active metabolites, such as Melatonin. To pursue this technological development, we have developed a UHPLC-MS/MS method to monitor 14 Tryptophan, Tryptamine, amino-benzoic, and pyridine metabolites. In addition, different extraction procedures to improve the recovery of Tryptophan and its derivatives from the vegetal matrix were tested. We investigated soybeans, pumpkin seeds, sesame seeds, and spirulina because of their botanical diversity and documented healthy effects. Four different extractions with different solvents and temperatures were tested, and water extraction at room temperature was chosen as the most suitable procedure to extract the whole Tryptophan metabolites pattern (called by us “indolome”) in terms of ease, high efficiency, short time, low cost, and sustainability. In all plant matrices, Tryptophan was the most abundant indole compound, while the pattern of its metabolites was different in the diverse plants extracts. Overall, 5-OH Tryptamine and Kynurenine were the most abundant compounds, despite being 100–1000-fold lower than Tryptophan. Melatonin was undetected in all extracts, but sesame showed the presence of a Melatonin isomer. The results of this study highlight the variability in the occurrence of indole compounds among diverse food plants. The knowledge of Tryptophan metabolism in plants represents a relevant issue for human health and nutrition.

## 1. Introduction

For several decades the benefits of the dietary styles abundant in fruit, vegetables, and legumes have been emphasized in terms of longevity, healthy ageing, and low morbidity from degenerative diseases, such as type 2 diabetes, metabolic syndromes, cardiovascular disease, neurodegenerative and neurobehavioral disorders, and some types of cancers. As an example, the Mediterranean diet can be considered the archetype of a health-promoting lifestyle, including moderate physical activity, a high intake of low-fat dairy products and healthy lipids (from seafood), as well as low consumption of refined sugars, red meat, and saturated fats [1,2].

The huge phytochemical diversity of plant foods is one of the reasons why well-balanced vegetable-based diets consistently afford health benefits. Plants typically contain hundreds of phytochemicals arising from different secondary metabolic pathways and contribute to plant defense from competitors and pests [3]. Varieties that humans selected for food use have lower contents of toxic and off-tasting phytochemicals and a higher content of bulk and micronutrients, especially of vitamins, essential fatty acids, amino acids, and antioxidants. Phenylpropanoids, a class of widespread phenylalanine derivatives including polyphenols (flavonoids, stilbenes, and proanthocyanidins), have been the most investigated dietary phytochemicals in the last decades, thus representing the paradigm of the relationship between food and health [4,5].

**Figure 1:**
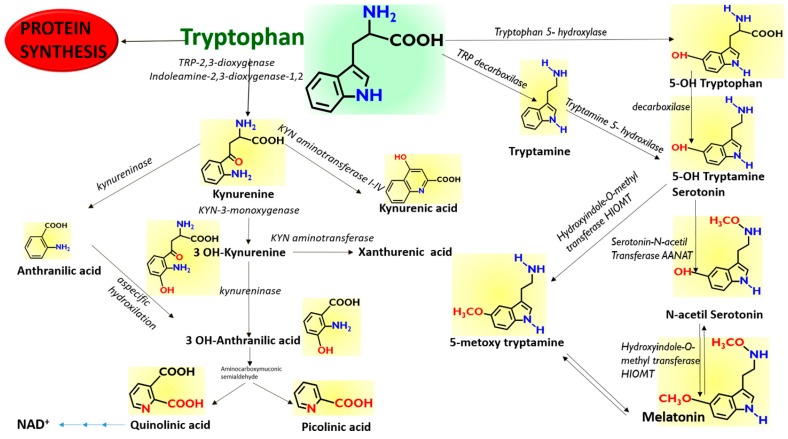
Scheme of the proposed pathways of Tryptophan. Tryptophan can be metabolized along the Kynurenine pathway along three different branches to Anthranilic acid, 3-OH Kynurenine, and Kynurenic acid, depending to the enzymatic repertoire of the cells. Tryptophan can also be metabolized along the Melatonin pathway through the synthesis of 5-OH Tryptamine (Serotonin) and by using preferentially different enzymes depending on the cellular repertoire.

The amino acid Tryptophan (TRP) is an irreplaceable constituent of the human diet, since the human genome does not include the enzymes that are necessary for its de-novo biosynthesis, but only those that perform some biotransformations and those responsible for break-down of the indole nucleus (Figure 1). An important class of neuro-transmitters, such as Serotonin, is derived from TRP, the source of which is only in the diet. A further biotransformed derivative of Serotonin, Melatonin (MEL), is also an important neurohormone, with multiple biological effects that include the circadian regulation of sleep. 

Recently, we suggested that indole compounds, in particular MEL (a potent neurohormone produced in traces from TRP in the pineal gland of mammals), discovered in some typical Mediterranean foods, such as wine and nuts, may represent a new element in this scenario, further corroborating the protective effects of diets rich in plant products [6,7,8]. 

In addition, the chemical feature of indole is widely represented in the phytochemicals’ structures, which range in complexity from simple derivatives of the amino acid TRP to the high chemical diversity of the huge family of indole alkaloids. 

Of note in plants, phenylalanine and TRP share a common biosynthetic route together with tyrosine, the shikimic acid or aromatic amino acid pathway unique of plants [3]. According to the USDA Food Composition Database (https://ndb.nal.usda.gov/ndb/), the sources that contain very high levels of free TRP include soy seeds (0.68 g TRP/100 g), pumpkin seeds (0.58 g TRP/100 g), and spirulina (0.93 g TRP/100 g).

Some studies have pointed out the ability of *Saccharomyces cerevisiae* and other high producer yeast strains to produce MEL by biotrasformation of TRP. In mammals, MEL is involved in many physiological functions: the regulation of circadian rhythms and sleep-wake cycle, immunostimulation, antioxidant defense, and tumor biology. Plants produce MEL with some similar functions to increase stress resistance, regulate circadian rhythms, and promote seed germination and fruit maturation. 

In this context, with the aim of using plants naturally rich in TRP as a substrate for yeast fermentation, we developed a multicomponent analytical method to measure in plant matrices 14 compounds that include the almost complete biotransformation pathways of TRP. Among these, nine have an intact indole nucleus and five have an oxidized open indole ring. We needed to specifically adapt suggestions from several published methods to fit the unique needs of our compound panel and examined plant matrices. Among the improvements is the choice of internal standards adapted for each compound class (amino acids, indoleamines, *N*-acetyl indoleamines, amino-benzoic, and pyridine derivatives). Following a well-trodden pathway, we propose “indolome” as the collective identifier for all metabolites in the TRP pathway, irrespective of whether they retain the indole nucleus or are converted to pyridine by the pyrrole-opening Kynurenine pathway and further recyclization.

This method has been applied to measure indole phytochemicals in soybeans, pumpkin seeds, sesame seeds, and spirulina, selected according to their botanical diversity and documented healthy effects. Different procedures were also tested in order to improve the metabolite extraction from each plant matrix.

This method will be useful for improving the knowledge on TRP-related compounds in plant foods and their biotransformation pathways. Moreover, it will help to select the most suitable plant source of TRP to be used as a natural substrate for yeast strains working as a “cell factory” for the production of antioxidants or bioactive compounds used for the production of “functional foods”.

## 2. Results and Discussion

### 2.1. Analytes Characterization by Fragment Ion Spectroscopy

To identify the best fragmentation transitions for use in detection by multiple reaction monitoring, a solution of each standard compound was directly infused into the ion source of the mass spectrometer with a built-in Hamilton syringe pump at a rate of approximately 10 µL/min. Typical conditions were exemplified for TRP. A 4 g/L (0.77 mol/L = 770 µM) water solution of TRP was diluted 1:1000 (770 nM) with an ESI ionization solvent of 50% methanol/water, containing 0.1% formic acid.

ESI source spectra were continuously scanned in the Q1 from 100 to 600 *m*/*z* in 2 s, and the relevant source parameters were optimized for the best signal-to-noise of the expected compound-related signals and optimal mass peaks shape and signal stability. The *Ramp* function of the Analyst software was employed to scan the declustering potential (DP) voltage of the instrument over values typically ranging from 0 (diffusion driven ion injection) to approximately 70 to 90 eV (voltage driven ion injection).

Collision induced fragment ion spectra of source-generated protonated molecules and first generation fragments were recorded by continuously scanning the Q3 mass filter over the relevant *m*/*z* range for the expected fragments and precursor ion. For fragmentation studies, gas pressure in the Q2 collision cell was selected at the low instrument setting. The *Ramp* function of the Analyst software was employed to scan the collision energy (CE) voltage of the instrument over values typically ranging from 0 (decomposition of metastable precursors) to approximately 50 to 70 eV (highest collision energy that yields reasonable ion current and structurally meaningful fragments). The integrated, transmission-convoluted array of spectra was acquired as the “integrated fragment ion spectrum” of each examined ion precursor. As an example, Figures S1–S4 show the integrated fragment spectra of protonated TRP, TRP D5, MEL, and MEL OCD3. Fragment abundance curves were extracted from the instrument data file as txt arrays of the fragment abundance vs. collision voltage from the Analyst software. All further elaboration was performed in custom Microsoft Excel spreadsheets, essentially according to Rubino et al. [9,10]. Figure S5 shows the elaboration of the fragment abundance curve for MEL and TRP (an operational surrogate of the “breakdown curve”, adapted to the specific task) to obtain the collision energy corresponding to the maximum yield of the selected fragment ion.

The optimization of MS/MS conditions as reported above yielded the values of the relevant mass spectrometry parameters that are reported in Table 1 where, in addition to retention time, two independent MRM transitions (quantifier and qualifier) for each analyte are also reported.

To ensure specificity, however, we used the data-dependent acquisition coupling MRM with EPI (enhanced product ion) (MRM-EPI). When the MRM is triggered, the ion trap records the full spectra (MS-MS) for that precursor, so providing a confirmative spectrum of the parent ion generating it. We recorded the on-the-fly fragment ion spectra to confirm peak identity for each analyte in each matrix. To apply this procedure in a routine fashion is, however, unfeasible, and the use of more than one MRM transition was enough.

### 2.2. Analytical Performances

**Figure 2:**
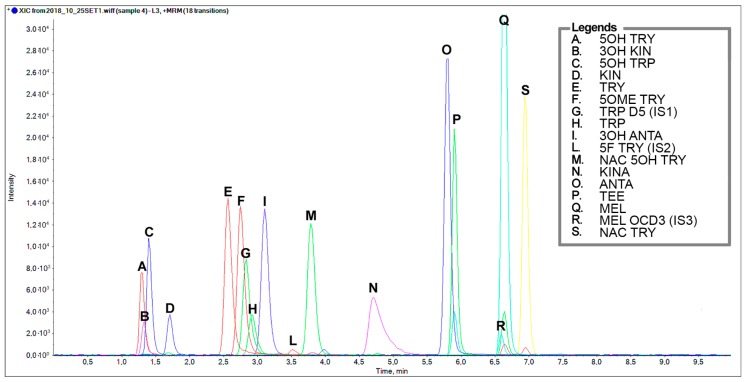
Representative chromatographic separation of a standard mixture of TRP-related metabolites containing an indole structure at a concentration 2 µg/mL, except for the internal standards: Tryptophan D_5_, 0.4 µg/mL; 5-F Tryptamine 20 ng/mL; Melatonin OCD_3_, 22 ng/mL. A. 5-OH Tryptamine, B. 3-OH Kynurenine, C. 5-OH Tryptophan, D. Kynurenine, E. Tryptamine, F. 5-OCH3 Tryptamine, G. Tryptophan D_5_, H. Tryptophan, I. 3-OH Anthranilic acid, L. 5-F Tryptamine, M. *N*-Ac-5-OH Tryptamine, O. Anthranilic acid, P. Tryptophan ethyl ester, Q. Melatonin, R. Melatonin OCD_3_, S. *N*-Ac Tryptamine.

Figure 2 shows the final chromatographic separation of a standard mixture of the indole compounds (2 µg/mL for all analytes except for internal standards which are 0.4 µg/mL TRP D5, 20 ng/mL 5F TRY, and 22 ng/mL MEL OCD3). MEL levels were previously assessed in almond and pistachios by using an LC-MS/MS incorporating the isotopomer analogue, MEL OCD3, which ensured a specific fragmentation and an improved performance for quantification [8].

Analytical performances were evaluated by studying parameters such as specificity, precision, accuracy, linearity, limit of detection (LoD), and limit of quantification (LoQ) (Table 2). The specificity was assessed by extracting water and water added in IS in each validation run. The lack of interfering peaks at the retention times of the analytes was considered as a sign of an acceptable selectivity. Precision and accuracy were calculated using different replicates of samples on the same day (intraday) and in different working days (interdays). Accuracy was expressed as the relative error (RE%), while precision was measured as a coefficient of variation (CV%). A CV% and RE% below 15% were considered suitable. Accuracy and precision were determined at low (5 ng/mL), medium (500 ng/mL), and high (10,000 ng/mL) concentrations in sets of three replicates per day. Results of precision and accuracy are expressed as means. Ten-point calibration curves were exactly constructed in different ranges so as to match the expected concentration of each singular analyte in the plant matrix (1–50,000 ng/mL). Linearity was assessed by unweighted least squares regression and expressed by the coefficient of determination (R^2^). LoD was considered as the concentration that generated a signal-to-noise ratio greater than 3 and LoQ was the lowest concentration that yielded a signal-to-noise ratio greater than 10 [11].

The pre-analytical treatment of plant extracts used before LC-MS/MS analysis was based solely on proteins removal by methanol extraction. The use of HybridSPE-Phospholipids cartridges (Sigma-Aldrich) to remove the phospholipid’s ion-suppression as described by Alvarez-Fernandez et al. [12] in yeast extracts, gave a loss on the TRP D5 recovery of more than 70%, so we decided to skip that passage. The samples’ treatment here described gave a process efficiency (peak area extracted samples/peak area unextracted samples) of >65%, considering both matrix effect and extraction efficiency, as calculated on the internal standard area (Table 3).

### 2.3. Extraction Efficiency and Indolome Profile in Different Food Plants

**Figure 3:**
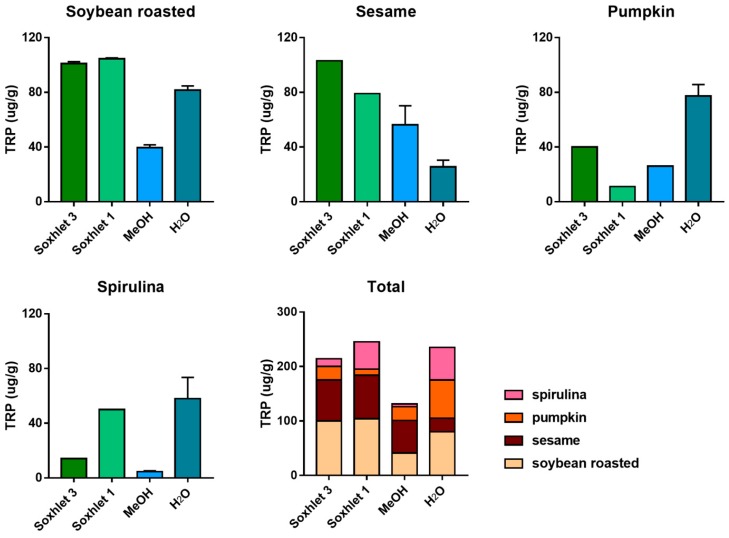
TRP extraction efficiency among four different procedures: defatting with petroleum ether and dichloromethane and successive extraction with methanol in a Soxhlet apparatus (Soxhlet 3), extraction in a Soxhlet apparatus with methanol only (Soxhlet 1), methanol extraction at room temperature (MeOH), and water extraction at room temperature (H_2_O). Data are presented as mean ± SD. The graph titled “Total” summarizes the efficiency of TRP extraction among plant materials and extraction procedures.

**Figure 4:**
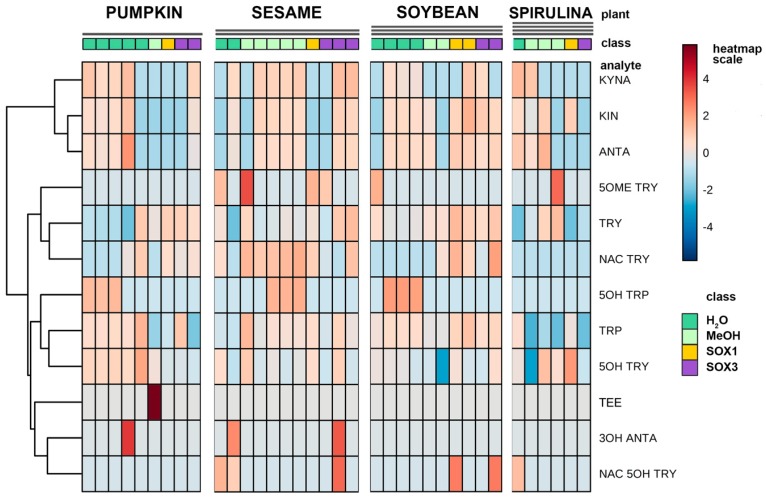
Hierarchical clustering heatmap representation of the concentration of the indolic compounds of four different plant matrices taking into consideration the different type of extractions (green: water extraction at room temperature; light green: methanol extraction at room temperature; yellow: extraction in a Soxhlet apparatus with methanol only, and violet: defatting with petroleum ether and dichloromethane and successive extraction with methanol in a Soxhlet apparatus). The concentrations were autoscaled and log-transformed for visualization. The color-scale differentiates values as high (red), mid (grey) and low (blue).

A number of different extraction procedures are available for extraction of bioactive phytochemicals from plant matrices [13,14]. The extraction efficiency of indole compounds from plants is highly dependent on the chemical structure, the different composition of vegetal matrices, and the extraction procedure used. Figure 3 shows the yield of TRP extraction from four different vegetal matrices, using four different extraction methods both at high and at room temperature. It is evident that the extraction with methanol at room temperature is not suitable for most of the plant materials used in this study, while the use of a Soxhlet apparatus at high temperature and with solvents of increasing polarity greatly improved the recovery. This was also shown when methanol alone was used at high temperature (Soxhlet 1). Despite these observations, the extraction procedure based on the use of water at room temperature was chosen as the reference method due to its low cost, sustainability, high efficiency of extraction, and short time needed. Except for sesame flour, water yielded TRP recovery in the same order of hot methanol. In Figure 3, the graph titled “Total” summarizes the efficiency of TRP extraction among all plant materials and extraction procedures. All the main indole compounds related to the TRP metabolism, the “indolome”, were investigated, and the results obtained by each vegetal matrix after being log-transformed and autoscaled are reported in Figure 4 as a heatmap and in Figure S6 as a PCA plot. From Figure S6 the chemical diversity of spirulina and sesame with respect to the other two matrices is evident. From the heatmap (Figure 4) water-based extraction was less efficient for 5OME TRY, TRY, NAC TRY in spirulina and pumpkin, while it gave a higher recovery for sesame and soybean. KINA, KIN, and ANTA were also well extracted from almost all matrices by water at room temperature.

We were interested in a relative comparison among different extraction procedures on 14 analytes that are very different in polarity, molecular weight, and concentration. Of course, some of them could benefit from the hot Soxhlet extraction, while others were well extracted at room temperature without solvent. In the end, we concluded that water is a sufficiently strong extractant for indolome analytes that are less polar than TRP, such as the MEL analogues, and we chose this extraction with the best safety and sustainability profiles to deepen our study.

**Figure 5:**
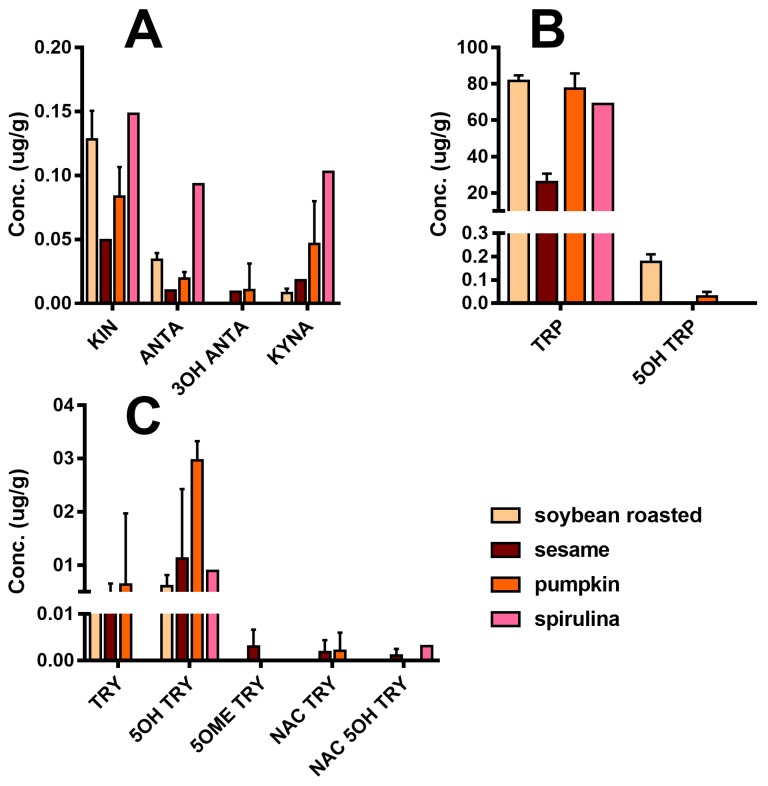
Indole compounds profile of plant extracts taking into consideration the water extraction at room temperature (concentrations are expressed as µg/g of plant materials ± SD): (**A**) Compounds derived from TRP catabolism, (**B**) TRP related compounds, and (**C**) Substituted tryptamines.

Figure 5 reports the detailed quali-quantitative composition of the indolome in the four vegetal sources after water extraction at room temperature. TRP is the most abundant compound in all the plant extracts, while 5OH TRP accounts for ≤1% in soybean and pumpkin and was undetectable in the other plants. Spirulina had the highest concentration of KIN, KINA, and ANTA with respect to the other matrices, whereas 3OH ANTA was undetectable. In sesame, all the TRP metabolites derived from the activity of tryptophane-2,3-dioxygenase (Figure 1) are present, including 3OH ANTA. In addition, sesame extraction provided the highest yield of metabolites derived from tryptophan decarboxylase or from tryptophan hydroxylase activity (TRY, 5OH TRY, and NAC TRY) and likely lead to MEL production. However, under the conditions used in these experiments, MEL was almost undetectable in all matrices. The specificity of our analysis was ensured by the use of the deuterated internal standard MEL OCD3, which confirmed the absence of signals in the trace of MEL [MH]^+^ 233.2 > 174.2, at the retention time of MEL OCD3 (236.2 > 177.2 *m*/*z*). In any case, we cannot exclude the presence of MEL in these matrices at pg/mL trace levels lower than our LoQ (2 ng/mL). The use of a specific purification by SPE can yield an improved LoQ < 80-fold (data not shown).

### 2.4. A Possible Melatonin-Interfering Compound in Sesame

**Figure 6:**
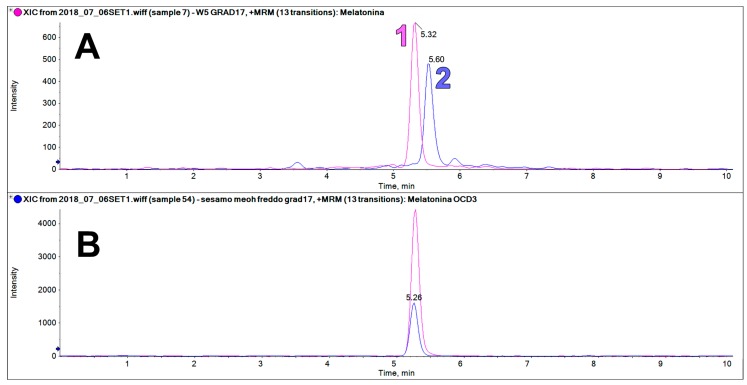
(**A**) LC-MS/MS analysis on the trace of MEL 233 > 174 *m*/*z* showing the peak of the MEL standard (1, RT 5.32, 62.5 ng/mL) and the peak of MEL isomers found in the sesame extract (2, RT 5.60). (**B**) LC-MS/MS analysis on the trace of MEL OCD3 (236 > 177 *m*/*z*) showing similar chromatographic behavior to the MEL standard.

The sesame extracts contain an intense chromatographic peak on the trace at 233.2 > 174.2 *m*/*z* at a retention time very close to that of MEL (5.32 vs. 5.60 min, Figure 6). This peak was only found in sesame flour, but not in all the other examined matrices. This component could be erroneously identified as MEL if it is not properly resolved chromatographically from the authentic MEL peak, or when a deuterated internal standard of MEL is not used.

**Figure 7:**
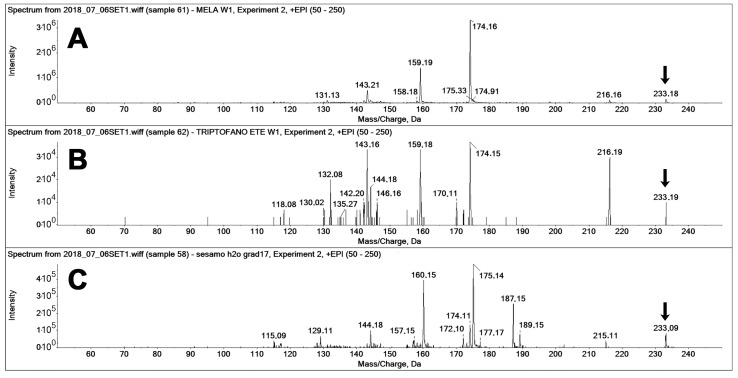
MS/MS spectra of different compounds with 233 *m*/*z* acquired on-the-fly in enhanced product ion (EPI) mode (at 20 eV collision energy). (**A**) Spectra of authentic MEL (standard solution, 20 ng injected), (**B**) Spectra of authentic TEE (standard solution, 20 ng injected), and (**C**) Spectra of MEL isomers in the sesame extracts (peak 2 in Figure 6). The latter presented a main 187 *m*/*z*, which is not appreciable on the spectra of the two standards.

The use of MRM-EPI was crucial for excluding false positives on the MEL transition. Figure 7 reports the fragment ion spectra recorded “on-the-fly” in the enhanced product ion (EPI) mode for the three compounds, namely, pure MEL, pure TEE (a known MEL isomer [15]), and the spectra of the unknown peak 2 found in the sesame extract (Figure 6). The constitutional isomer TEE [15] is a known interfering compound in the analysis of MEL, the retention time of which is, in our system, sufficiently different as to separately identify the two compounds, even if their fragment spectra feature the same fragments, although with different relative intensities (Figure 7A,B).

The spectrum of the unknown peak (Figure 7Cfeatures the same precursor ion of MEL (233 *m*/*z*) but features two main fragments that are up-shifted by one *m*/*z* unit (160 and 175 *m*/*z*) from those of MEL and TEE (159, 174 *m*/*z*). Two further signals, at 187 and 189 *m*/*z*, are present only in the spectrum of the unknown peak.

It is difficult to speculate on the identity of the component that yielded peak A2 (Figure 6), due to the high complexity of the sesame metabolome [16]. However, a literature search shows that sesame contains a specific lignan, sesamolin (C_20_H_18_O_7_; MW 370), a non-nitrogenous asymmetrical compound, the mass spectra of which contain a fragment at 233 *m*/*z* [17,18]. This fragment can originate from low critical energy elimination processes para to a methylenedioxy aromatic system further bearing an aryl-alkyl ether, and yielding a 1,2,4-trioxy-benzene neutral fragment. This decomposition can occur through a charge-remote 1,2- or 1,6-rearrangement of a protonated or ionized precursor (Figure S7). Depending on the desolvatation and declustering conditions of the ESI ionization used in our analytical method, in-source decomposition may produce the first-generation transition to 233 *m*/*z* from sesamolin. However, the spectrum of sesamolin published by Dar et al. [17] (Figure 4B of reference), and reportedly acquired under ESI ionization, shows several differences with respect of that expected based on standard fragmentation rules of even-electron protonated molecules [19,20]. In fact, the published spectrum does not show the expected protonated molecule (371 *m*/*z*), but does show signals at 369 and 370 *m*/*z* that may be compatible with the molecular cluster of a [M − H]^+^ species, but not with the expected MH^+^. The reported spectrum also displays much more abundant fragmentation than seemingly compatible with “soft” ESI ionization, and the supplied instrumental details do not allow for a judgement on the energy of the processes in the recording of the spectrum. In addition, the observed fragments are likely derived from the overlap of odd-electron and even-electron decomposition pathways. Due to the current unavailability of a sesamolin standard, it is not possible to confirm or disprove the identification of the observed unknown peak in the sesame extract.

## 3. Materials and Methods

### 3.1. Reagent and Chemicals

The chemicals were all analytical grade and purchased by Sigma-Aldrich (Milan, Italy). All aqueous solutions were prepared using purified water in a Milli-Q (Millipore, Milan, Italy) device. Melatonin (MEL), 5-F Tryptamine (5F TRY; IS1),Tryptamine (TRY), 5-OH Tryptamine (5OH TRY), 5-OCH_3_ Tryptamine (5OME TRY), *N*-Ac Tryptamine (NAC TRY), *N*-Ac-5-OH Tryptamine (NAC 5OH TRY), Tryptophan-D_5_ (TRP D5; IS2), 3-OH Anthranilic acid (3OH ANTA), 3-OH Kynurenine (3OH KIN), Anthranilic acid (ANTA), Kynurenic acid (KINA), Kynurenine (KIN), Tryptophan (TRP), Tryptophan ethyl-ester (TEE), and 5-OH-Tryptophan (5OH TRP) standards was purchased from Sigma-Aldrich (Milan, Italy). The isotopomer Melatonin-OCD_3_ (MEL OCD3) was synthesized by Prof. Andrea Penoni (Dipartimento di Scienza e Alta Tecnologia, Università dell’Insubria, Varese).

### 3.2. Preparation of Standard Solution

Standard stock solutions of the analytes were prepared individually at a concentration 1 mg/mL in water, except for 3OH ANTA and KINA, which were dissolved in methanol/water 1:1. The mixture of internal standards (MIX IS) were prepared as described: 20 µL of each solution, 2 mg/mL TRP D5, 10 µg/mL 5F TRY, and 11 µg/mL MEL OCD3 brought to the final volume of 10 mL with methanol 0.5% formic acid. The stock solutions and the MIX IS (0.4 µg/mL TRP D5, 20 ng/mL 5F TRY, and 22 ng/mL MEL OCD3) were stored at 4 °C.

### 3.3. Plant Material

Organic flours from soybean, pumpkin, and sesame seeds were purchased from Fior di Loto and Molino Bongiovanni companies (Turin, Italy). A suitable specimen of each was dried in a ventilated oven set at 50 °C for 24 h before extraction. Organic spirulina was kindly provided by Spireat (Cremona, Italy), and pulverized in a mortar before use.

### 3.4. Extraction Procedure from Plant Samples 

Various techniques were applied in extraction of the plant sample constituents in order to compare their efficiency. All procedures were carried out in dim light conditions to prevent metabolite degradation by photochemical reactions. 

#### 3.4.1. Soxhlet Extraction

Each sample (5 g) was weighed into a cellulose thimble and placed in a Soxhlet apparatus equipped with a 0.25 L round bottom flask and a water-cooled condenser. A three-step extraction was performed using solvents of increasing polarity (150 mL), i.e., petroleum ether (40 °C), dichloromethane (60 °C), and methanol (65 °C) for 8 h (4–6 cycles h^−1^) (Soxhlet 3) [13,14]. In the Soxhlet apparatus, both the flours and the Spirulina were also subjected to one-step extraction using only methanol without previous defatting by nonpolar solvents (Soxhlet 1). All obtained extracts were stored at −20 °C until analysis

#### 3.4.2. Methanol Extraction at Room Temperature

Methanol (3 mL/g) was added to flours and alga. The obtained mixtures were stirred for 20 min after 10 min sonication. Then, each sample was decanted and the supernatant centrifuged at 28,000 rpm for 10 min. The resulting extract was filtered through a 0.20 μm filter and combined with that of a second extraction before storage at −20 °C.

#### 3.4.3. Aqueous Extraction at Room Temperature

Distilled water (3 mL/g) was added to flours and alga. The obtained mixtures were stirred for 20 min after 10 min sonication. Then, each sample was decanted and the supernatant centrifuged at 28,000 rpm for 10 min before storage at −20 °C.

### 3.5. Purification of Plant Extracts

A small aliquot of plant extract (10 µL) was added with 50 µL of MIX IS (20 ng TRP D5, 1 ng 5F TRY, and 1.1 ng MEL OCD3) and 100 µL of precipitating solution (methanol + 0.5% formic acid). After vortexing, 100 µL of solution was withdrawn and evaporated until dry. Then, it was reconstituted with 50 µL water + 0.5% formic acid, and 10 µL was directly injected in the LC-MS/MS.

### 3.6. LC-MS/MS Analysis for the Study of Indolic Compounds

The analytical system consisted of an HPLC coupled to a tandem mass spectrometer. The liquid chromatograph system was a Dionex 3000 UltiMate instrument with autosampler, binary pump, and column oven (Thermo Fisher Scientific, Waltham, MA, USA). Separation of 17 indolic compounds (14 analytes and 3 internal standards) was attained on a reversed-phase Zorbax Eclipse XDB-C18 (4.5 × 50 mm, 1.8 µm) analytical column preceded by a security guard cartridge. Linear gradient was obtained by mixing eluent A (water + 0.1% formic acid) and eluent B (methanol + 0.1% formic acid). The elution gradient was set as follows: 0–1 min (20% B), 1–5 min (20%–60% B), 5–7 (60% B), 7.0–7.2 (60%–95% B), 7.2–8.2 (95% B), 8.2–8.5 (95%–5% B), 10 (5% B). The flow rate was 0.4 mL/min and the column temperature was 40 °C. The tandem mass spectrometer was an AB Sciex 3200 QTRAP instrument with electrospray ionization TurboIonSpray™ source (AB Sciex, Milano, Italy). Instruments were managed with the proprietary manufacturer’s software and according to the manufacturer’s instructions. The analytical data were processed using Analyst software (version 1.2). The ion spray voltage was set at 5.5 kV and the source temperature was set at 500 °C. Nitrogen was used as a nebulizing gas (GS 1, 55 psi), turbo spray gas (GS 2, 60 psi), and curtain gas (30 psi). The collision-activated dissociation (CAD) was set to a low level. The dwell time was set at 70 ms, and the MS scan was performed in positive ion modes (ESI+). MS/MS experiments were conducted using nitrogen as the collision gas. Compound-dependent parameters were optimized via direct infusion. Multiple reaction monitoring (MRM) mode was used with a fixed dwell time of 60 ms for all transitions and analytes. Quantitative analysis was performed interpolating each peak area of analyte/area internal standards (IS) with calibration curves of each indole compounds. 

## 4. Conclusions

TRP is an essential amino acid, which cannot be synthesized de novo in animals, but rather is only obtained from food sources. Therefore, the knowledge of TRP metabolism in food plants can be of some interest for the researcher and professionals involved in the nutritional and health-promoting assessment of plant foods [21]. We found water extraction at room temperature the most sustainable and suitable for our scopes. In the future, depending also on the indole compounds of interest, it will be interesting to assess how exhaustive is this extraction, and/or to test alternative and innovative procedures [22].

In particular, the use of LC-MS/MS, as above described, allows for the elucidation of chemical composition and content in micronutrients and nutraceutical components, and may improve awareness on the chemical diversity and nutritional value of food plants.

## Figures and Tables

**Table 1:** Conditions for LC-MS/MS analysis of indole species.

Name	Abbreviation	RT	MW	DP	Q1	Q3(1)	CE(1)	Q3(2)	CE(2)
**5-F Tryptamine (IS1)**	**5F TRY**	**3.89**	**178**	**20**	**179.2**	**162.2**	**15.2**	**115.0**	**36**
Tryptamine	TRY	2.89	160	20	161.2	144.2	14.8	117.0	33.5
5-OH Tryptamine	5OH TRY	1.30	176	20	177.2	160.2	14.7	115.0	30
5-OCH_3_ Tryptamine	5OME TRY	3.13	190	20	191.2	174.2	14.3	130.0	48
*N*-Ac Tryptamine	NAC TRY	6.87	202	20	203.2	144.2	19.1	117.0	39
*N*-Ac-5-OH Tryptamine	NAC 5OH TRY	3.71	218	20	219.2	160.2	18.5	115.0	43
**Tryptophan D_5_ (IS2)**	**TRP D5**	**3.03**	**209**	**20**	**210.2**	**192.2**	**14.8**	**150.0**	**24.5**
3-OH Anthranilic acid	3OH ANTA	2.91	153.1	21	154.1	135.9	17	80.0	35
3-OH Kynurenine	3OH KIN	1.30	224.2	16	225.1	208.1	13	110.2	21
Anthranilic acid	ANTA	5.63	137.1	21	138.1	119.8	15	65.0	39
Kynurenic acid	KINA	4.56	189.1	36	190.1	89.1	51	163.0	25
Kynurenine	KIN	1.72	208	21	209.2	192.2	15	94.0	19
Tryptophan	TRP	3.12	204	20	205.2	188.2	13.4	118.0	35
Tryptophan ethyl ester	TEE	6.06	232	20	233.2	216.2	13.1	132.0	34
5-OH Tryptophan	5OH TRP	1.60	220	16	221.1	204.2	15	161	25
**Melatonin OCD_3_ (IS3)**	**MEL OCD3**	**6.52**	**235**	**20**	**236.2**	**177.2**	**19.1**	**131**	**42**
Melatonin	MEL	6.56	232	20	233.2	174.2	18.8	131	42

Each subclass is preceded by the correspondent internal standard (IS) (in bold) used for quantification. Q3(1), CE(1) refer to the quantifier ions, whereas Q3(2), CE(2) refer to the qualifier ions. RT retention time, DP declustering potential, MW molecular weight, Q1 first quadrupole, Q3 third quadrupole, CE collision energy.

**Table 2:** Analytical performances. Some relative error (RE%) values (in bold) were >15%, but were still considered suitable for our purposes.

Analytes	LoQ	LoD	Slope	Intercept	R^2^	Precision		Accuracy	
	(ng/mL)	(ng/mL)				Intra	Inter	Intra	Inter
Kynurenine	50	15	0.0002	−0.0828	0.995				
*Low*						14.0	15.0	15.0	14.8
*Medium*						4.5	9.0	15.0	13.8
*High*						11.3	9.4	8.8	6.4
Anthranilic acid	5	2	0.0011	−0.1047	0.998				
*Low*						7.2	13.3	14.9	16.0
*Medium*						14.2	12.6	13.6	12.4
*High*						5.0	7.3	3.4	5.6
3-OH Anthranilic acid	20	5	0.0008	−0.5867	0.995				
*Low*						7.0	15.5	14.4	19.8
*Medium*						8.1	14.4	15.0	13.1
*High*						2.8	11.4	2.0	9.9
Kynurenic acid	25	8	0.0006	−0.1483	0.998				
*Low*						7.0	9.1	11.5	9.8
*Medium*						2.3	3.7	7.5	5.0
*High*						5.0	5.7	4.0	3.9
3-OH Kynurenic acid	50	15	0.0001	−0.0713	0.996				
*Low*						11.1	14.6	13.9	18.7
*Medium*						7.6	14.0	10.1	14.1
*High*						3.5	8.4	2.6	7.2
Tryptamine	10	3	0.0065	0.9310	0.993				
*Low*						1.7	3.3	12.7	16.2
*Medium*						4.7	4.5	10.4	13.9
*High*						3.7	4.3	3.1	3.3
5-OH Tryptamine	20	7	0.0027	0.5467	0.995				
*Low*						14.8	12.2	14.9	12.8
*Medium*						3.2	8.7	4.9	10.7
*High*						5.5	5.7	3.8	4.1
5-OCH_3_ Tryptamine	10	3	0.0086	1.0595	0.998				
*Low*						13.4	13.5	14.7	11.6
*Medium*						3.4	5.8	13.2	12.8
*High*						2.7	4.1	2.0	3.3
*N*-Ac Tryptamine	5	1	0.0071	1.7840	0.996				
*Low*						0.9	3.4	19.0	19.9
*Medium*						3.7	5.5	18.9	19.9
*High*						4.6	4.1	3.1	4.0
Tryptophan	15	4	0.0002	−0.1059	0.996				
*Low*						12.0	13.6	15.0	12.2
*Medium*						9.3	6.7	7.9	5.9
*High*						4.5	3.1	3.5	9.3
*N*-Ac-5-OH Tryptamine	10	4	0.0050	0.5555	0.996				
*Low*						5.3	8.4	19.0	16.9
*Medium*						4.4	5.5	18.7	17.4
*High*						7.4	10.9	5.1	8.3
Tryptophan ethylester	10	4	0.0007	−0.0211	0.999				
*Low*						13.0	9.9	14.5	14.6
*Medium*						7.6	9.1	8.0	9.2
*High*						5.5	3.9	4.1	3.0
Melatonin	2	0.5	0.0088	0.8414	0.999				
*Low*						5.4	3.7	14.6	14.7
*Medium*						7.5	5.0	14.8	11.3
*High*						1.3	4.9	1.1	3.1
5-OH Tryptophan	20	5	0.0004	−0.1296	0.997				
*Low*						10.8	10.3	13.0	14.9
*Medium*						8.5	7.7	11.8	9.5
*High*						6.4	6.3	4.9	4.6

**Table 3:** Process efficiency (peak area of IS extracted/peak area of IS not-extracted) of internal standards among the different plant extracts.

Plant Materials	5F TRY	TRP D5	MEL OCD3
Soybean extract	65	67	77
Pumpkin extract	69	73	75
Sesame extract	104	83	68
Spirulina extract	70	82	71

## Supplementary Materials

The following are available online at https://www.mdpi.com/1420-3049/25/2/311/s1, Figure S1: Integrated spectra MS/MS of Tryptophan, Figure S2: Integrated spectra MS/MS of Tryptophan D5, Figure S3: Integrated spectra MS/MS of Melatonin, Figure S4: Integrated spectra MS/MS of Melatonin OCD3, Figure S5: Operational surrogate of the “breakdown curve” of (A) Melatonin *m*/*z* 233 > 174, (B) Melatonin OCD3 *m*/*z* 236 > 177, (C) Tryptophan *m*/*z* 205 > 188 and (D) Tryptophan D5 *m*/*z* 210 > 192, Figure S6: PCA plot with an explained variance of 52.1% and 19.6% on principal components 1 and 2, respectively, Figure S7: Possible mechanism underlying the formation of the 233 *m*/*z* fragment from in-source decomposition of the protonated parent ion 371 *m*/*z* of sesamolin.

## Abbreviations

Tryptamine (TRY), *N*-Ac Tryptamine (NAC TRY), 5-OH Tryptamine or Serotonin (5OH TRY), 5-OCH_3_ Tryptamine (5OME TRY), *N*-Ac-5-OH Tryptamine (NAC 5OH TRY), 5-F Tryptamine (5F TRY; IS1), 5-OH Tryptophan (5OH TRP), Tryptophan (TRP), Tryptophan D_5_ (TRP D_5_; IS2), Tryptophan ethyl ester (TEE), Anthranilic acid (ANTA), 3-OH Anthranilic acid (3OH ANTA), Kynurenic acid (KYNA), Kynurenine (KYN), 3-OH Kynurenine (3OH KIN), Melatonin or *N*-Ac-5- OCH_3_ Tryptamine (MEL), Melatonin OCD_3_ (MEL OCD_3_, IS3), solid phase extraction (SPE), retention time (RT), declustering potential (DP), molecular weight (MW), first quadrupole (Q1), third quadrupole (Q3), collision energy (CE).
