# Supplementary material for: LC-MS/MS-Based Profiling of Tryptophan-Related Metabolites in Healthy Plant Foods
Source: Molecules. 2020 Jan 13;25(2):311. doi: 10.3390/molecules25020311 (PMC7024187; doi:10.3390/molecules25020311)
Supplement: Supplementary file 1 [file molecules-25-00311-s001.zip › molecules-670533-SI.pdf]

LC-MS/MS-Based Profiling of Tryptophan-Related Metabolites in Healthy Plant Foods

Supplementary Material

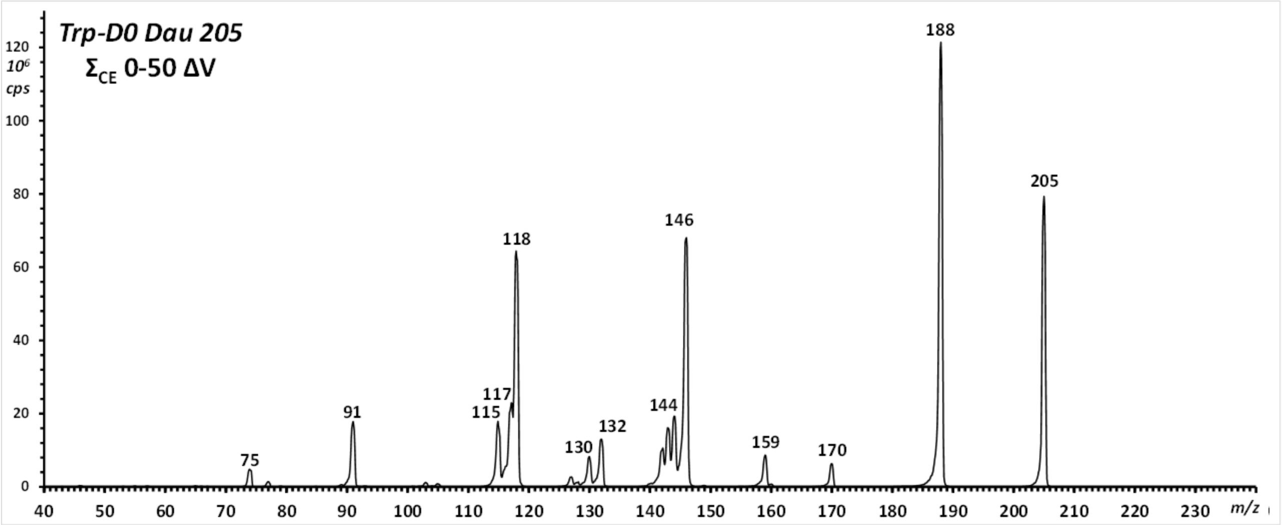

Figure S1. Integrated spectra MS/MS of Tryptophan.

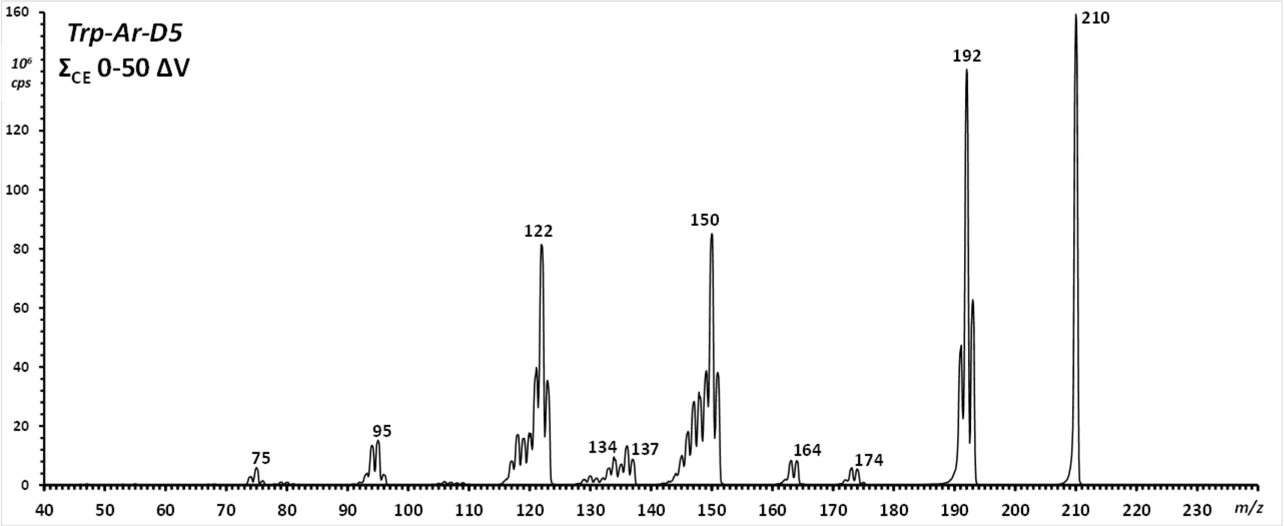

Figure S2. Integrated spectra MS/MS of Tryptophan D5.

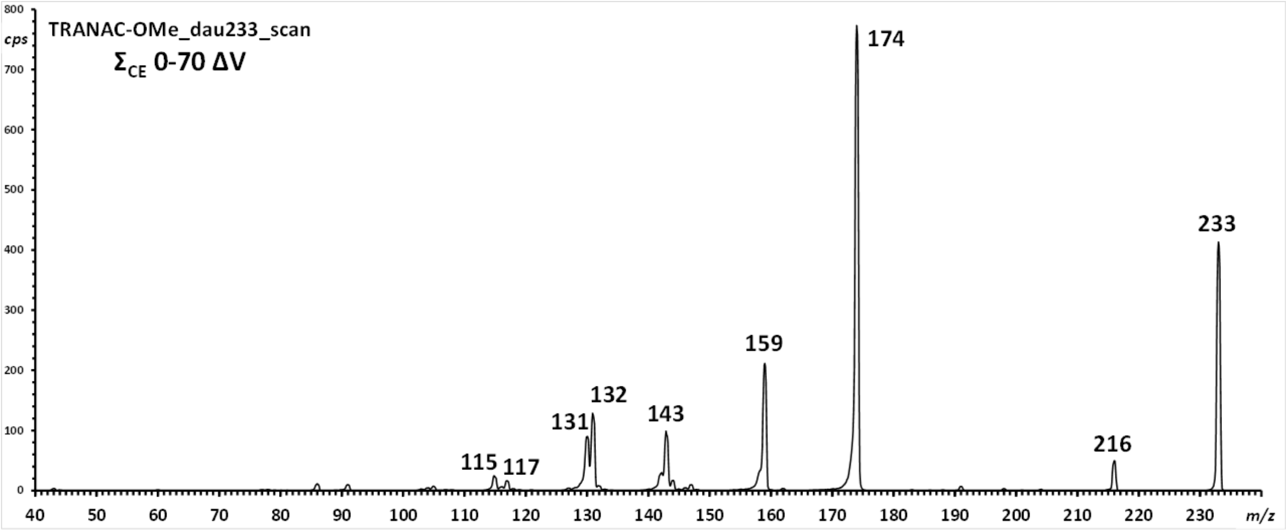

Figure S3. Integrated spectra MS/MS of Melatonin.

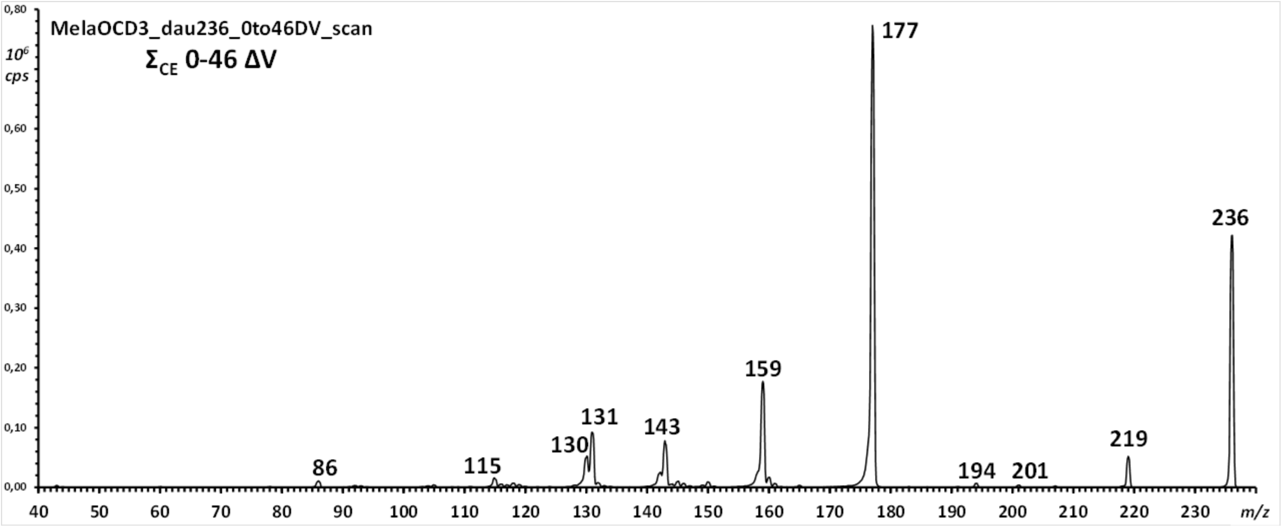

Figure S4. Integrated spectra MS/MS of Melatonin OCD3.

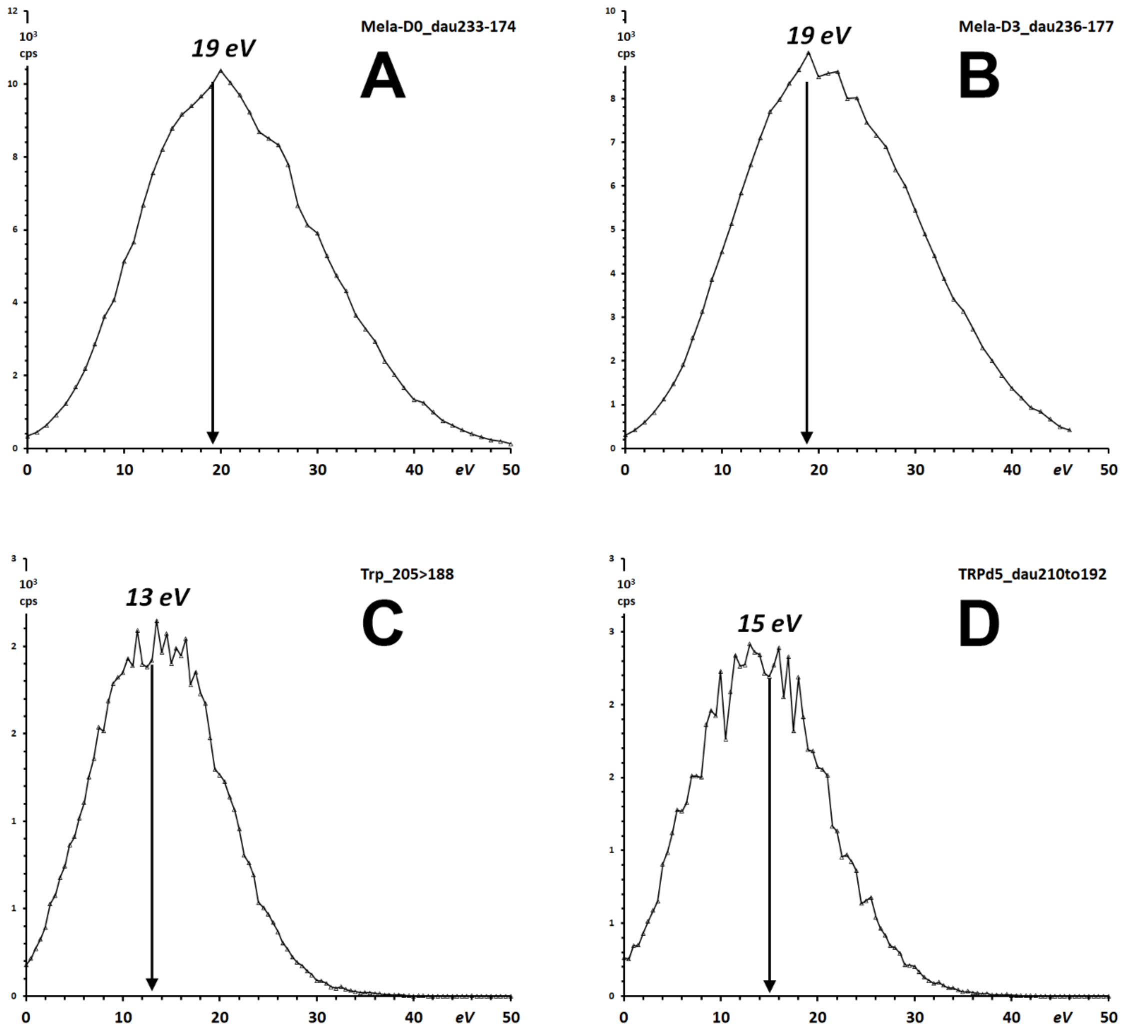

Figure S5. Operational surrogate of the "breakdown curve" of (**A**) Melatonin *m/z* 233 > 174, (**B**) Melatonin OCD3 *m/z* 236 > 177, (**C**) Tryptophan *m/z* 205 > 188 and (**D**) Tryptophan D5 *m/z* 210 > 192. They were adapted to the specific task to obtain the collision energy corresponding to the maximum yield of the selected fragment ion.

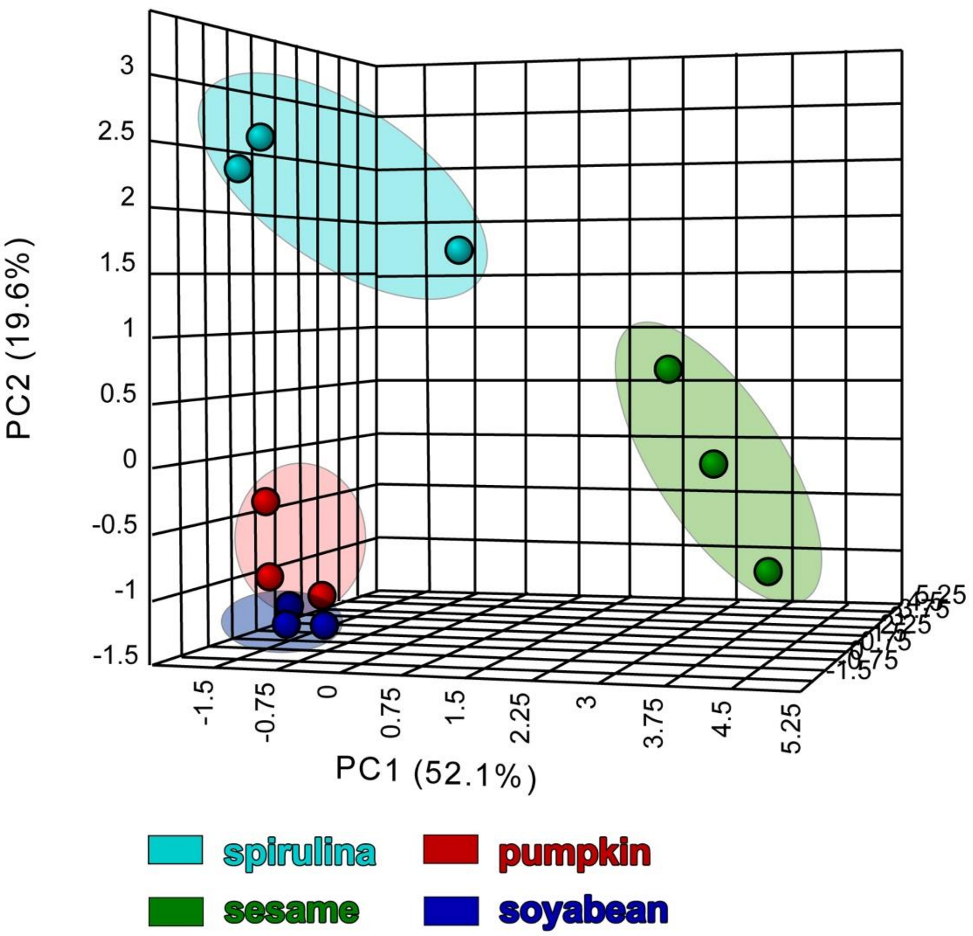

Figure S6. PCA plot with an explained variance of 52.1% and 19.6% on principal component 1 and 2, respectively. Data represents the indolome composition of different plant materials using the same water-based extraction protocol.

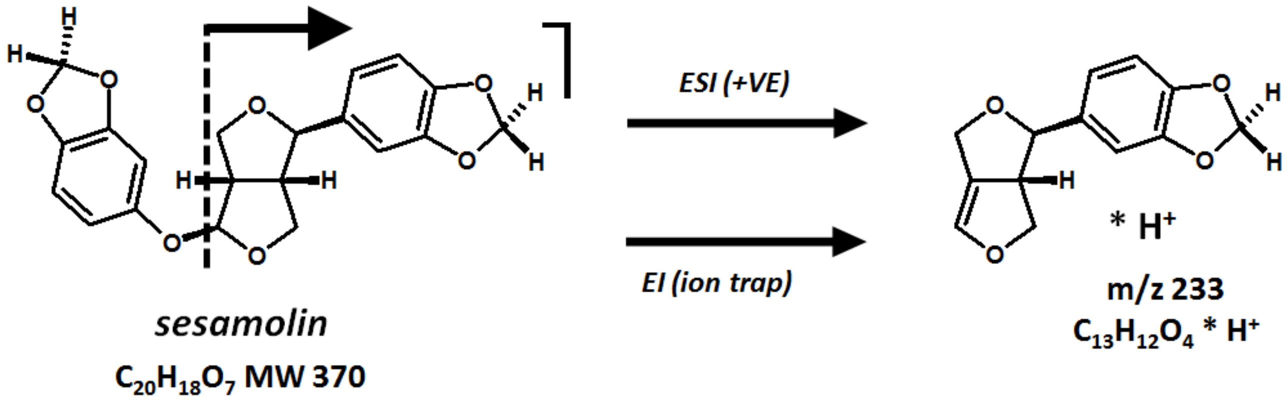

Figure S7. Possible mechanism underlying the formation of the *m/z* 233 fragment from in-source decomposition of the protonated parent ion *m/z* 371 of sesamolin. This fragment can originate from low critical energy elimination processes para- to a methylenedioxy aromatic system further bearing an aryl-alkyl ether, and yielding a 1,2,4-trioxy-benzene neutral fragment. This decomposition can occur through a charge-remote 1,2- or 1,6- rearrangement of a protonated or ionized precursor. .
